# Immunization with a novel Clostridium perfringens epsilon toxin mutant rETX^Y196E^-C confers strong protection in mice

**DOI:** 10.1038/srep24162

**Published:** 2016-04-06

**Authors:** Wenwu Yao, Jingjing Kang, Lin Kang, Shan Gao, Hao Yang, Bin Ji, Ping Li, Jing Liu, Wenwen Xin, Jinglin Wang

**Affiliations:** 1 State Key Laboratory of Pathogen and Biosecurity, Institute of Microbiology and Epidemiology, Beijing 100071, PR China

## Abstract

Epsilon toxin (ETX) is produced by toxinotypes B and D of *Clostridium perfringens*. It can induce lethal enterotoxemia in domestic animals, mainly in sheep, goats and cattle, causing serious economic losses to global animal husbandry. In this study, a novel and stable epsilon toxin mutant rETX^Y196E^-C, obtained by substituting the 196th tyrosine (Y196) with glutamic acid (E) and introducing of 23 amino acids long C-terminal peptide, was determined as a promising recombinant vaccine candidate against enterotoxemia. After the third vaccination, the antibody titers against recombinant wild type (rETX) could reach 1:10^5^ in each immunized group, and the mice were completely protected from 100 × LD_50_ (50% lethal dose) of rETX challenge. The mice in 15 μg subcutaneously immunized group fully survived at the dose of 500 × LD_50_ of rETX challenge and 80% of mice survived at 180 μg (1000 × LD_50_) of rETX administration. *In vitro*, immune sera from 15 μg subcutaneously immunized group could completely protect MDCK cells from 16 × CT_50_ (50% lethal dose of cells) of rETX challenge and protect against 10 × LD_50_ dose (1.8 μg) of rETX challenge in mice. These data suggest that recombinant protein rETX^Y196E^-C is a potential vaccine candidate for future applied researches.

Epsilon toxin (ETX) is synthesized as a prototoxin by toxinotypes B and D of *Clostridium perfringens*^1^,^2^. The prototoxin has no or poor activity, and is activated when its 23 C-terminal residue is cleaved by proteases^3^. ETX is a pore-forming toxin and plays a key role in enterotoxemia, primarily in sheep, goats, cattle and other livestock, which can cause severe economic losses^4^,^5^. Because the activated ETX is one of the most known potent toxins, it is classified as a category B biological agent by USA Centers for Disease Control and Prevention (CDC)^6^,^7^,^8^.

An effective way to prevent ETX-induced enterotoxemia in domestic animals is to produce vaccines^1^. Over the past decades, vaccines have been largely based on toxoids or formaldehyde-treated bacterial culture filtrates^9^. These vaccines pose several problems: it is hard to completely remove formaldehyde, inflammatory responses after vaccination and the immunogenicity of epsilon toxin are variable^9^,^10^. Therefore, it is essential and urgent to develop innovative vaccines against enterotoxemia. Amino acid substitution of a protein toxin is a good way to generate a mutant, which is also a promising way to develop a toxin mutant vaccine. It is crucial to identify key amino acid residues of ETX to generate variants with low toxicity and while retaining the immunogenicity.

Previous studies showed that ETX contains three structural domains (I, II, III), and there are some key amino acid residues including Tyr29, Tyr30, Tyr36, Tyr196 and Phe199 in domain I, which is considered to play pivotal in binding to the receptor^11^,^12^,^13^. Domain II, which contains a β-hairpin is believed to be involved in membrane insertion^14^. Domain III, which contains C-terminal peptide, is likely to play a role in monomer-monomer interaction^12^. In addition, our previous studies have shown that the C-terminal peptides and the Y196E amino acid mutation synergistically and largely alleviate the toxicity of ETX^15^. Therefore, toxin mutant with a Y196E substitution and a C-terminal peptide is more likely to be a promising vaccine candidate.

In this study, our work has focused on testing the immunogenicity and other features of the candidate vaccine: rETX^Y196E^-C. Previous studies have proved that epsilon toxin can cross the blood-brain barrier and accumulate in the brain, binding to endothelial cells, causing degeneration and necrosis, and increasing vascular permeability and perivascular edema^16^,^17^. However, only a few cell lines are susceptible to the epsilon toxin including madin-darby canine kidney (MDCK) cells and human leiomyoblastoma (G-402) cells^16^,^18^. The MDCK cell line is most frequently used in epsilon studies, which, is why we chose the MDCK cells to determine the neutralizing activity of antibody *in vitro*. The evaluation results showed that rETX^Y196E^-C is a promising vaccine candidate.

## Materials and Methods

### Animals

All animal experiments were approved by the Animal Ethics Committee of the Academy of the Military and Medical Sciences, and were performed in accordance with the Guideline for Animal Experiments of the Academy of Military and Medical Sciences.

### Expression and purification of rETX^Y196E^-C

rETX (without the 13 N-terminal and 23 C-terminal amino acid residues) and rETX^Y196E^-C (without the 13 N-terminal sequences and containing an Y196E mutation) were purified using a nickel-nitrilotriacetic affinity chromatography (GE Healthcare, Piscataway, NJ, USA) as previously described^19^,^20^.

### Thermal stability

Thermal stability assay was performed according to the manufacturer’s instructions of the Protein Thermal Shift Dye Kit (Applied Biosystems) to further evaluate the recombinant toxins rETX^Y196E^-C and rETX. 12.5 μL of toxins (0.8 mg/mL) were mixed with 5 μL of Protein Thermal Shift Buffer and 2.5 μL of Diluted Protein Thermal Shift Dye (8×), and the fluorescence and melting temperature (T_m_) were obtained using the StepOnePlus Real-Time PCR system (Applied Biosystem) with a 1% thermal gradient from 37 °C to 99 °C.

### Immunization with rETX^Y196E^-C

Six-week-old female BALB/c mice (about 18–22 g), purchased from Laboratory Animal Center of The Academy of Military Medical Sciences, were divided into seven groups (twenty mice of each group) randomly. These groups of mice were immunized by two immune pathways: subcutaneous injection and intraperitoneal injection, and different concentrations of rETX^Y196E^-C: 5 μg/mouse, 10 μg/mouse, 15 μg/mouse and 0.01M phosphate-buffered saline solution (PBS). Protein rETX^Y196E^-C was diluted with PBS and mixed with the same volume of Freund’s adjuvant. The mice were immunized three times and each vaccination was performed at an interval of two weeks. Serum was collected from the tail vein of mice one week after each immunization. The survival status and weight changes were recorded daily.

### Measurement of antibody titers

Enzyme-linked immunosorbent assay (ELISA) was used to measure the sera antibody titers of immunized BALB/c mice. The 96-well plate was coated with 100 μL of 10 μg/mL rETX in 0.05 M carbonate-buffered saline solution overnight, and then the plate was blocked with 200 μL of 5% bovine serum albumin (BSA) for 2 h at 37 °C. After being incubated with 100 μL of 10-fold serial diluted serum for 1 h at 37 °C and washed three times by PBST (0.01M PBS, 0.05% Tween-20), the plate was incubated with HRP-coupled goat anti-mouse IgG (1:50000) at 37 °C for 1 h. Then the plate was washed three times with PBST and added the TMB substrate solution (TIANGEN Biotechnology) to each plate, and finally 2 M H_2_SO_4_ was used to stop the reaction. The absorbance was determined at 450 nm by a microplate reader (Molecular Device).

### Assessment of antibody affinity

ELISA was used to assess the affinity between rETX^Y196E^-C-induced antibody and rETX or rETX^Y196E^-C according to previous studies^21^. The proteins rETX or rETX^Y196E^-C (10 μg/mL) were coated into the 96-well plate overnight at 4 °C and washed three times with PBS. The plate was blocked with 5% BSA diluted in 0.01M PBS for 1 h at 37 °C. The 2-fold diluted rETX^Y196E^-C immunized sera (come from 15 μg/mouse subcutaneous immunized mice), PBS immunized sera (1:100) or mouse anti-ETX monoclonal antibody (developed by our laboratory) were added to the plate and incubated at 37 °C for 1 h. After being washed three times with PBST, the plate was incubated with NH_4_SCN diluted in PBS at 37 °C for 15 min at the following concentrations of 8.0 M, 4.0 M, 2.0 M, 1.0 M, and 0.5 M. The plate was washed three times and the HRP-coupled goat anti-mouse IgG (1:50000) was added to the plate and incubated at 37 °C for 1 h. The absorbance was obtained by a microplate reader.

### Challenge with rETX

One week after the third immunization, mice immunized by rETX^Y196E^-C were challenged with 1000 × LD_50_, 500 × LD_50_, and 100 × LD_50_ of rETX and mice immunized with PBS were challenged with 10 × LD_50_ of rETX intraperitoneally, the value of LD_50_ (180 ng/mouse) comes from our previous paper^15^. The survival status of all mice was daily recorded for 5 days.

### *In vitro* neutralization assay

rETX (50 μL) at doses of 1 × CT_50_ (440 ng/mL), 2 × CT_50_, 4 × CT_50_, 8 × CT_50_, 16 × CT_50_ and 32 × CT_50_ was incubated with an equal volume of mouse polyclonal antiserum from mice of 15 μg/mouse subcutaneous groups or from immunized mice with PBS for 30 min at 37 °C. The toxin-antiserum mixtures were added to the 96-well plate seeded with 2–3 × 10^4^ MDCK cells and incubated at 37 °C for 24 h. After the plate was washed three times with PBS, 100 μL of culture medium containing 20 μL MTS was added to each well and incubated for 3 h. The absorbance was measured at 490 nm, which reflected the number of living cells.

The sera samples (500 μL) from 15 μg/mouse subcutaneous immunized mice were mixed with an equal volume of the doses of 10 × LD_50_, 100 × LD_50_ or 500 × LD_50_ rETX and incubated for 30 min at 37 °C. Groups of five female BALB/c mice were challenged intraperitoneally with 500 μL of the mixed solution. The survival status was observed daily for 5 days.

### Histopathologic alterations

Previous study indicated that ETX mainly accumulated in brains and kidneys of sensitive animals and caused sudden death^22^. Mice subcutaneously immunized three times with PBS and 15 μg of rETX^Y196E^-C were challenged with 1000 × LD_50_ of rETX. The brains and kidneys from challenged mice were separated for histopathologic study.

## Result

### Thermal stability of recombinant toxins

In this assay, the fluorescent dye could bind to exposed hydrophobic regions of the unfolded proteins and the fluorescent signal had a significant increase. The structure of protein changed with temperature, and the hydrophobic regions were exposed. Thus, the melting temperature, which is a characteristic of protein stability, can be automatically monitored by detecting the change in fluorescent signals. As shown in Table 1, the T_m_ of the rETX^Y196E^-C and rETX were 93.41 ± 0.23 °C and 88.60 ± 3.86 °C respectively. The results indicated that the stability of the mutant was similar to the wild toxin, and they were structurally similar.

### Specific antibody response induced by rETX^Y196E^-C

**Figure 1:**
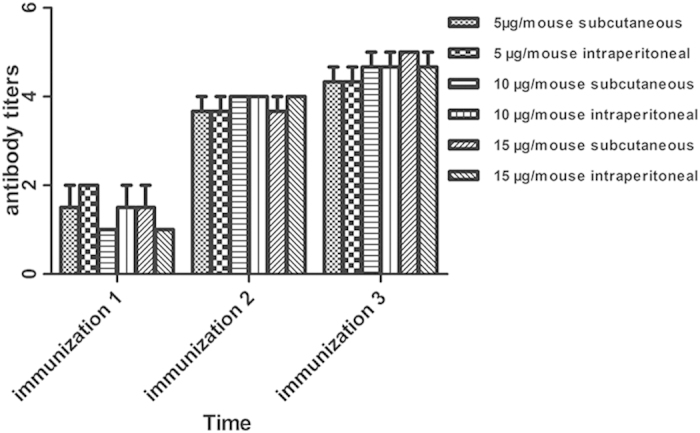
Antibody titers after each immunization. Mice were vaccinated three times and one week after each immunization the sera were collected from tail veins, then measure the antibody titers using ELISA. The arithmetic mean titers of 3 mice per group ± SD were shown in figure. ANOVA was used to analyze the results.

One week after each vaccination, the antibody titers were measured by ELISA. The data are shown in Fig. 1. The results showed that the antibody titers were 1:10^2^ after the initial immunization, but the antibody titers increased to 1:10^4^ after the second immunization. One week after the third vaccination, the mean IgG titer against protein rETX^Y196E^-C of the six groups could reach about 1:10^5^ and each group revealed similar titers and had no statistical differences (*P* > 0.05), which indicated that vaccination of mice with recombinant toxin rETX^Y196E^-C induced a specific antibody response.

### Antibody affinity

**Figure 2:**
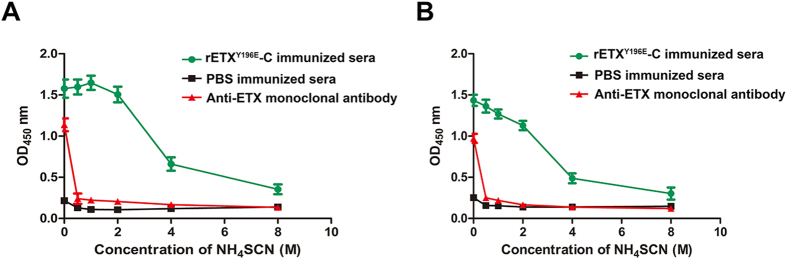
Measurement of antibody affinity. The affinity between antiserum and rETX (**A**) or rETX^Y196E^-C (**B**) was shown in graphs. The affinity indexes between antiserum and rETX or antiserum and rETX^Y196E^-C are 4.28 ± 0.77 M and 3.31 ± 0.75 M respectively.

The results of antibody affinity assay were obtained and shown in Fig. 2. The affinity indexes between antiserum and rETX (Fig. 2A) or rETX^Y196E^-C (Fig. 2B) were defined as the concentration of NH_4_SCN when the absorbance reached the half of the highest absorbance. Figure 2 indicated that the two affinity indexes were 4.28 ± 0.77 M and 3.31 ± 0.75 M respectively, and had no significant difference (*P* > 0.05), but they were higher than those of PBS immune group and anti-ETX monoclonal antibody group.

### Protective effect in mice immunized with rETX^Y196E^-C

Mice immunized with different doses and routes of rETX^Y196E^-C were challenged with 100, 500 and 1000 × LD_50_ of active rETX per mouse. The result showed that mutant rETX^Y196E^-C stimulated strong protection against enterotoxemia in mice. All mice survived in the six rETX^Y196E^-C immunized groups which were challenged with 100 × LD_50_ dose (Table 2), and all mice survived in the three rETX^Y196E^-C subcutaneously immunized groups which were challenged with 500 × LD_50_ dose (Table 2). Furthermore, 80% of the mice in the 15 μg/mouse subcutaneous group survived when the challenge dose increased to 1000 × LD_50_ of rETX (Table 2). However, Mice immunized with PBS died when challenged with 10 × LD_50_ dose of rETX (Table 2). In addition, those results indicated that immunized pathway and dose contributed greatly to protective effect. Subcutaneously immunized pathway was better than intraperitoneally immunized pathway, and 15 μg rETX^Y196E^-C was the most efficient dose to induce a specific antibody response and protect mice from ETX challenge.

**Figure 3:**
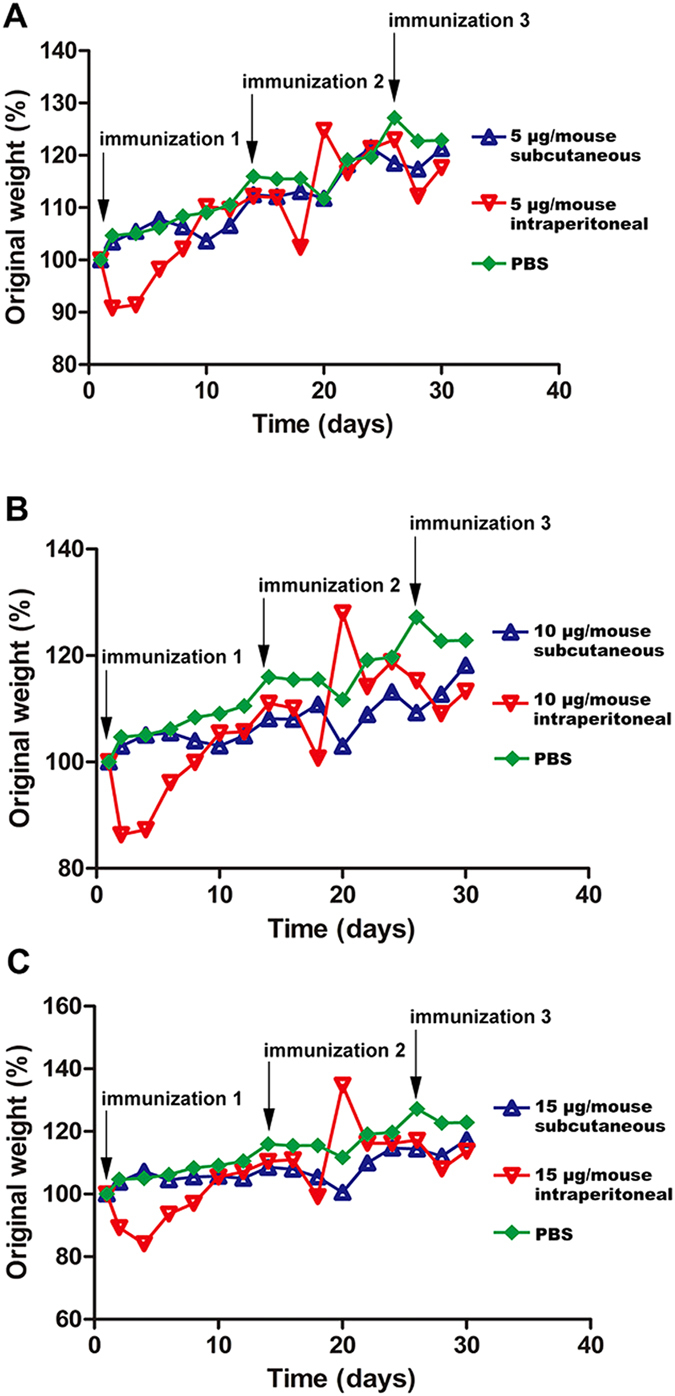
Average weight changes of immunized mice. The average weights of 5 μg (**A**), 10 μg (**B**), 15 μg (**C**) and PBS immune groups of mice were recorded every two days after the first immunization and the weights were recorded for 30 days (the arrows pointing the time of each immunization). Each point represents the current total weight relative to original total weight.

The body weights of mice in each group were recorded daily for 30 days, and the weight changes are shown in Fig. 3. After the initial immunization, the mean body weight of mouse in 15 μg/mouse lost about 20% of their body weight (Fig. 3C). Similarly, mice in 5 μg/mouse intraperitoneal group and 10 μg/mouse intraperitoneal group lost less than 20% of their body weight. However, the mean body weights of mouse in subcutaneous groups had slight or no changes (Fig. 3A,B). Those data showed that intraperitoneally immunized pathway was more toxic for mice than subcutaneously immunized pathway during the first vaccination.

### *In vitro* neutralization assay

#### Toxin neutralization assay in MDCK cells

**Figure 4:**
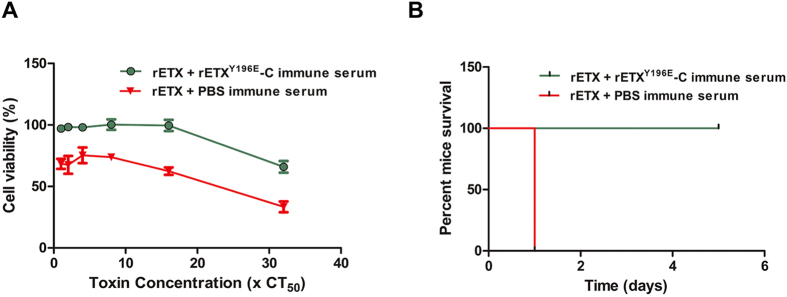
*In vitro* neutralisation assay. (**A**) Toxin neutralization assay in MDCK cells, 50 μL different concentration of the rETX (1×, 2×, 4×, 8×, 16× and 32 × CT_50_) was mixed with equal volume of antiserum for 30 min at 37 °C, the viability of cells were determined by MTS. The results were analysed by ANOVA and there is a significant difference between the viability rates of the two groups (*P* < 0.05). (**B**) Toxin neutralization assay in mouse, the mice were challenged with the mixture of 10 × LD_50_ and equal volume of antiserum, the survival curves is obtained by calculating the survival rates.

Antiserum in this assay was collected from mice in 15 μg/mouse subcutaneous group. The MDCK cells were incubated with toxin-antiserum mixtures, and the viability was obtained. The ETX neutralization effects occurred only in the sera from the rETX^Y196E^-C vaccinated mice. As shown in Fig. 4A, the serum was able to protect MDCK cells against wild type ETX. However, the sera from PBS vaccinated mice did not inhibit ETX induced cytotoxicity and the cell viabilities were significantly lower than those of rETX^Y196E^-C vaccinated groups (*P* < 0.05).

#### Toxin neutralization assay in mouse

To test the ETX neutralization effect *in vivo*, mice were injected with mixtures of activated rETX and antiserum, and the survival results are shown in Fig. 4B. All mice survived when challenged with mixtures of 10 × LD_50_ rETX and antiserum. The results indicated that the anti- rETX^Y196E^-C antibodies could neutralize 10 × LD_50_ dose of rETX and protect mice against ETX challenge, however, the mice died when the concentration increased to 100 × LD_50_ dose of rETX.

### Histopathologic analysis

**Figure 5:**
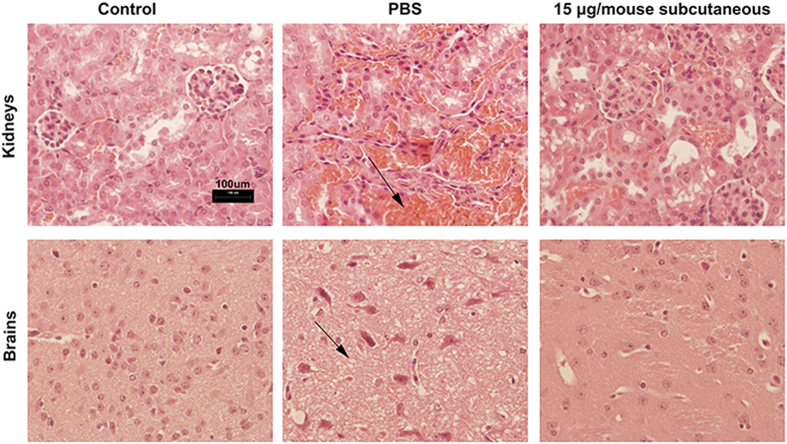
Histopathologic alterations in brains and kidneys of mice. In this graph, control group represents the normal mice, PBS group represents the PBS immunized mice challenged with 1000 × LD_50_ of rETX and 15 μg/mouse subcutaneous group represents the mice immunized subcutaneously with 15 μg of rETX^Y196E^-C challenged with 1000 × LD_50_ of rETX. In PBS group, obvious hemorrhage (Upper arrow) was occurred in kidney, and vacuolation and mild edema (Lower arrow) were occurred in brain. The situation was significantly improved in 15 μg/mouse subcutaneous group mice.

After being challenged with 1000 × LD_50_ dose of rETX, kidneys and brains of mice in PBS immunized group showed obvious pathological alterations including serious hemorrhage, cytoplasmic vacuolation and mild edema compared to that of the control group (Fig. 5). However, there were no phanerous pathological alterations in rETX^Y196E^-C immunized group (Fig. 5). The results showed that rETX^Y196E^-C immunization could protect the sensitive organs against harm induced by ETX.

## Discussion

Previous studies have reported that site-directed mutants of ETX could significantly reduce the toxicity and be considered vaccine candidates^1^,^9^,^20^. The mutant H106P has previously been reported to non-toxic to mice and the immunized mice could survive at the 1000 × LD_50_ dose of wild type ETX^9^. Recombinant epsilon toxoid vaccines could lead to a rise in serum potency in animals and as potential vaccine candidates to protect against enterotoxemia, but it took a long time to inactivate recombinant toxins^23^,^24^. Also, our team developed a mutant F199E, which showed a 2572-fold decrease in toxicity compared with that of wild type ETX and it can protect the immunized mice against a challenge of 100 × LD_50_ dose of rETX^20^. However, mutant F199E was unstable and was more likely to precipitate. Meanwhile, the expression level of the mutant F199E in the soluble form was relatively low. It was previously reported that the mutant Y196E showed a slight deficiency in toxicity, and one of our previous studies concerning the mutant Y196E had achieved similar results. However, we also found that the C terminal peptide and amino acid Y196E mutation synergistically alleviated the toxicity of ETX, the cytotoxicity of rETX^Y196E^-C (13,110 ng/mL) was more significantly reduced than that of rETX (440 ng/mL) and the LD_50_ of rETX^Y196E^-C (1,770,110 ng/kg) was reduced more than 200-fold compared with rETX (7910 ng/kg)^15^. In addition, mutant rETX^Y196E^-C showed a higher level of expression in the soluble form and a robust stability, which makes it easy to develop and store antigens. Therefore, rETX^Y196E^-C is more likely to be a promising vaccine candidate. Recently, mutant with two mutations Y30A-Y196A showed that it could markedly reduce cytotoxic activities in MDCK cells and was considered a recombinant vaccine candidate against enterotoxemia. However, the data of challenge of the immunized animal were not provided^1^.

Immunizing doses and inoculation routes were optimized to obtain a better protective effect. Although varied immunizing doses and inoculation routes have achieved similar antibody titers, the dose of 15 μg per mouse and the subcutaneous immunization have provided the best protection of mice. High dose inoculation and subcutaneous immunization are likely to raise the proportion of effective antibodies. Even if the dose of 15 μg per mouse may not be the best and a higher immunizing dose may strengthen the protection, it may also exhaust the immune system and do some harm to immunized mice. Besides, we found that subcutaneous immunization was a safer way than intraperitoneal immunization, since mice in three intraperitoneally immunized groups lost about 20% of their original body weight after the first vaccination (Fig. 3).

Toxoid vaccines for use in domesticated sheep and goats are widely available commercially and have been used extensively over the past decades^25^. Although toxoid vaccines are effective in preventing enterotoxaemia in animals, inflammatory responses following vaccination have been reported to lead to reduced feed consumption^26^. However, the mutant rETX^Y196E^-C showed a predominant safety. For one thing, rETX^Y196E^-C has poor toxicity to MDCK cells or mice. For another, the weight of subcutaneously immunized mice in vaccination period keeps increasing rather than decreasing, as in control groups, which suggested that administration of rETX^Y196E^-C did not interrupt the growth of the mice.

The mutant rETX^Y196E^-C possessed high immunogenicity. The mean IgG titers could reach about 1:10^5^ after the third vaccination. Besides, the affinity between antibodies and ETX was relatively high (Fig. 2). Accordingly, the mutant rETX^Y196E^-C could provide an excellent protective effect. Under the optimal condition, rETX^Y196E^-C could completely protect mice against challenge of 500 × LD_50_ dose of wild type rETX and protect 80% of mice against a dose of 1000 × LD_50_ of rETX. This protective effect is better than that of mutant F199E and may be better than that of mutant H106P, since our previous studies showed that H106P could provide similar protective effect as F199E. Previous studies suggested H106P could form the basis of a vaccine against ETX^9^. Therefore, rETX^Y196E^-C could be another promising vaccine candidate. Besides, immune sera stimulated by rETX^Y196E^-C could be considered an immunotherapeutic drug. The *in vitro* neutralization assay showed that 50 μL of immune sera was able to significantly reduce the cytotoxicity of 32 × CT_50_ dose of rETX to MDCK cells (*P* < 0.05) (Fig. 4), while mice could be completely protected against 10 × LD_50_ dose of rETX by 250 μL of immune sera without showing any clinical symptoms.

In conclusion, all these results in the current study demonstrate that rETX^Y196E^-C is a potential recombinant vaccine candidate against animal enterotoxemia. Further studies should be performed to determine whether rETX^Y196E^-C is able to protect susceptible animals, such as sheep, goats and calves, from enterotoxemia induced by epsilon toxin.

### Statistical analysis

Treatment group differences were analyzed using analysis of variance (ANOVA) and student’s paired t-test. *P < 0.05 represents statistical significance between the two groups.

## Additional Information

**How to cite this article**: Yao, W. *et al.* Immunization with a novel Clostridium perfringens epsilon toxin mutant rETX^Y196E^-C confers strong protection in mice. *Sci. Rep.* **6**, 24162; doi: 10.1038/srep24162 (2016).

## Figures and Tables

**Table 1:** The thermal stability of recombinant toxins.

Groups	T_m_ ± SD^a^
rETX	88.60 ± 3.86
rETX^Y196E^-C	93.41 ± 0.23

**Table 2:** The survival rates of the mice vaccinated with different concentration of rETX^Y196E^-C or PBS challenged with rETX.

Groups	Alive/total			
	100 × LD_50_	500 × LD_50_	100 × LD_50_	10 × LD_50_
5 μg/mouse subcutaneous	0/5	4/5	5/5	
5 μg/mouse intraperitoneal	0/5	0/5	5/5	
10 μg/mouse subcutaneous	0/5	5/5	5/5	
10 μg/mouse intraperitoneal	0/5	0/5	5/5	
15 μg/mouse subcutaneous	4/5	5/5	5/5	
15 μg/mouse intraperitoneal	0/5	1/5	5/5	
PBS			0/5	0/5
